# Dupilumab in Severe Asthma–COPD Overlap: Real-Life Experience on a Case Series

**DOI:** 10.3390/jpm16020108

**Published:** 2026-02-10

**Authors:** Bruno Sposato, Gianna Camiciottoli, Leonardo Gianluca Lacerenza, Elena Bargagli, Paolo Cameli, Giovanna Elisiana Carpagnano, Manuela Latorre, Elisa Petrucci, Valentina Fabbrini, Laura Giannini, Alberto Ricci, Andrea Serafini, Marco Scalese

**Affiliations:** 1 Pneumology Department, Local Health Unit ‘Toscana Sud-Est’, ‘Misericordia’ Hospital, 58100 Grosseto, Italy; 2 Severe Asthma Unit, Department Experimental and Clinical Biomedical Sciences “Mario Serio”, University of Florence, Careggi University Hospital, 50134 Florence, Italy; gianna.camiciottoli@unifi.it; 3 Department of Pharmaceutical Medicine, Local Health Unit ‘Toscana Sud-Est’, ‘Misericordia’ Hospital, 58100 Grosseto, Italy; leonardogianluca.lacerenza@uslsudest.toscana.it (L.G.L.); elisa.petrucci@uslsudest.toscana.it (E.P.); 4 Respiratory Diseases Unit, Department of Medical and Surgical Sciences & Neurosciences, Siena University Hospital, 53100 Siena, Italy; bargagli2@gmail.com (E.B.); paolo.cameli@yahoo.com (P.C.); 5 Institute of Respiratory Disease, Department of Translational Biomedicine and Neuroscience, University “Aldo Moro”, 70121 Bari, Italy; elisiana.carpagnano@uniba.it; 6 Pulmonary Unit, UOC Pneumologia Nord, Toscana Nord Ovest, Nuovo Ospedale Apuano, 54100 Massa, Italy; manuela.latorre@yahoo.it; 7 Corporate Clinical Trials Task Force, Clinical Research Office, Local Health Unit ‘Toscana Sud-Est’, San Donato Hospital, 52100 Arezzo, Italy; valentina.fabbrini@uslsudest.toscana.it (V.F.); laura1.giannini@uslsudest.toscana.it (L.G.); 8 Division of Pneumology, Department of Clinical and Molecular Medicine, Sapienza University of Rome, AOU Sant’Andrea, 00189 Rome, Italy; alberto.ricci@uniroma1.it; 9 Medical Management Department, Local Health Unit ‘Toscana Sud-Est’, ‘Misericordia’ Hospital, 58100 Grosseto, Italy; andrea.serafini@uslsudest.toscana.it; 10 Institute of Clinical Physiology, Italian National Research Council, 56124 Pisa, Italy; scalese@ifc.cnr.it

**Keywords:** asthma–COPD overlap, dupilumab, asthma, effectiveness

## Abstract

**Background/Objective**: Little is known about the efficacy of biologics and in particular Dupilumab in patients with severe asthma associated with COPD (SA-COPD) features. The objective of this study was to determine whether Dupilumab has similar clinical/functional efficacy in individuals with SA-COPD and in those with pure severe asthma (SA). **Methods**: We retrospectively selected 11 consecutive patients with SA with COPD features (smoking history of at least 15 pack/years; emphysema on chest CT scan; FEV_1_ < 80%; RV and TLC > 130%; DLCO < 70; salbutamol reversibility test < 12%) treated with Dupilumab for at least 1 year. These subjects were compared with 33 consecutive patients with SA alone who were also treated with the same biologic for at least 12 months. **Results**: FEV_1_ and FEF_25–75_ changes after treatment were 10 ± 18.3% and 18.6 ± 26.5% in the SA group, whereas they were 4.8 ± 7.6% and 7.2 ± 6.8% in individuals with SA-COPD (*p* = 0.909 and *p* = 0.102 respectively). Similarly, ACT (5.3 ± 3.1 vs. 5.6 ± 3.7; *p* = 0.783) and exacerbation changes (−2.97 ± 1.3 vs. −4 ± 4.3; *p* = 0.960) after Dupilumab were similar in the two groups. No differences were also found in FeNO and BEC changes (−18 ± 22 vs. −21.3 ± 21.1 ppb and −63.6 ± 415 vs. −142 ± 299 cells/µL respectively; *p* = 0.984 and *p* = 0.481). The percentages of subjects that reduced and stopped OC therapy and those that stepped down the level of ICS dose after treatment were also similar in the two populations. After adjustment for multiple confounding factors, changes in all evaluated outcomes also remained comparable between patients with SA-COPD and those with SA. **Conclusions**: In our experience, Dupilumab is effective both in patients with SA alone and in those with asthma–COPD overlap. We must always consider T2 inflammation in the management of such patients in order to provide the most appropriate treatment.

## 1. Introduction

Uncontrolled asthmatics requiring oral corticosteroids for frequent exacerbations, despite receiving maximal maintenance therapy with a high dose of inhaled corticosteroids (ICSs) combined with long-acting beta-2 agonist (LABA) and anticholinergic (LAMA) bronchodilator medications (whose correct inhalation technique has been determined) and exhibiting a good adherence to treatment, with comorbidities and aggravating factors under control, are considered to be affected by an asthma phenotype defined as “severe” [1]. This condition, especially if associated with T2 inflammation, may require additional treatment with a biologic. In fact, depending on the levels of inflammatory biomarkers such as blood eosinophils, IgE, and FeNO, along with low lung function and persistent disease exacerbations, we can combine maintenance therapy with various biologics such as omalizumab, mepolizumab, benralizumab, dupilumab, and tezepelumab.

Severe asthma may be associated with chronic obstructive pulmonary disease (COPD), especially in patients with significant smoking history [2,3]. The GINA and GOLD guidelines introduced the concept of asthma–COPD overlap (ACO) in 2014 [1,4], and various definitions of ACO have been reported [5,6,7,8]. The term “asthma–COPD overlap” was used to describe cases with both the features of asthma and COPD characterized by mixed airway inflammation. According to the GINA and GOLD guidelines, patients usually have chronic airway disease symptoms with cough, wheezing, recurrent lower respiratory tract infection, and asthma and COPD features combined [1,4]. In general, the diagnostic criteria for ACO obviously include a significant smoking history, age, presence of persistent airway obstruction, incomplete airway reversibility, eosinophilic inflammation, atopy, and increased IgE levels. The prevalence of ACO has been reported to vary between 13.3% and 61.0% [9,10,11,12]. Such variability depends on the diagnostic criteria used and on the characteristics of the study population, such as age, medications taken, smoking status, and asthma severity. Two recent studies have demonstrated that approximately 27–30% of patients with severe asthma (SA) had characteristics consistent with ACO [2,3].

Previous studies have shown that patients with ACO had a higher symptom burden, a worse quality of life, experience more frequent exacerbations, and displayed lower lung function than those with asthma alone [2,12,13,14]. Among patients with SA, those with ACO used more systemic corticosteroids and had more frequent exacerbations related to emergency department visits [2]. Therefore, the presence of COPD features is recognized as a clinical indicator of worsening and as a negative prognostic factor.

Little is known about the real-life efficacy of biologics in patients with SA-COPD overlap. Given the greater severity of the overlapping of these two conditions, it is conceivable that biologics, including Dupilumab, may be less effective in subjects with SA associated with COPD. Since there are few studies in the literature on this topic, the effect of biologics in severe ACO is not quite clear. In fact, a recent study has highlighted that patients with ACO treated with biologics exhibited worse outcomes than asthma patients [15], while another study has found no differences in the effectiveness of biologics in ACO and pure asthma [16]. In particular, there is no clear evidence regarding the efficacy of Dupilumab (an anti-interleukin-4/13) in asthma–COPD overlap to date. Dupilumab has been recently approved for treatment in COPD patients who have type 2 inflammation, as indicated by elevated blood eosinophil counts, because, when treated with this biologic, they showed fewer exacerbations, better lung function and quality of life, and less severe respiratory symptoms than those who received a placebo [17,18]. A recent real-life study also showed that the use of Dupilumab was associated with lower rates of exacerbations and improved symptoms in patients with COPD and elevated blood eosinophils [19]. Therefore, it is conceivable that Dupilumab may be at least as effective in subjects with asthma alone as in patients with severe asthma complicated by COPD.

Considering this, we aimed to analyze whether Dupilumab may have a different effectiveness in subjects with pure asthma compared to individuals with SA-COPD in real life.

## 2. Materials and Methods

In the period from 1 January 2023 to 31 December 2024, we retrospectively searched our database (digitized data extracted from medical records and spirometers) for 11 consecutive patients with severe asthma and COPD overlap (SA/COPD) who had been treated with dupilumab for at least 12 months and 33 consecutive patients (in a 1:3 ratio) with severe asthma (SA) alone who had also been treated with dupilumab for at least 1 year. The following outcomes obtained in such two groups after treatment with Dupilumab were compared: changes in FEV1% predicted, FEF_25–75_% predicted, Asthma Control Test (ACT), exacerbations (by subtracting pre-biologic from post-biologic values) and the number of subjects that took low/medium/high doses of inhaled corticosteroids (ICSs), as well as the number of individuals who achieved oral corticosteroid (OC) sparing after Dupilumab. 

All patients had been poorly controlled even while using high ICS doses, long-acting bronchodilators, anti-leukotrienes (montelukast) and/or OCs, which made it necessary to add Dupilumab, as recommended by step 5 of the GINA asthma guidelines [1]. Dupilumab was prescribed to subjects that showed a peripheral blood eosinophil count ≥ 150/μL and/or fractional exhaled nitric oxide (FeNO) ≥ 25 that were continuously or frequently treated with OCs before the first Dupilumab dose. Information was collected for each patient regarding allergic sensitization (to *Dermatophagoides pteronyssinus* and *D. farinae*, grass mix, *Parietaria*, *Olea europaea*, *Cupressus sempervirens*, *Betula pendula*, *Alternaria tenuis*, *Aspergillus* spp., dog and cat dander, and others), serum IgE levels, blood eosinophil counts, and the presence of rhinitis, sinusitis, nasal polyposis, and/or other comorbidities (such as systemic hypertension, chronic heart disease, diabetes, osteoporosis, gastroesophageal reflux, COPD, obesity, and others), as well as age, smoking habits, and body mass index (BMI). Furthermore, asthma onset age and treatment period were also recorded. Lung function variables (FEV_1_%, FEF_25–75_%, RV%, TLC%, DLCO%), ACT, blood eosinophil counts (BECs), fractional exhaled nitric oxide (FENO) and the number of moderate/severe exacerbations (that required at least 3 days of OC treatment) were evaluated at the time of Dupilumab prescription and at the end of each patient’s treatment period. ICS doses, OC use/doses, Montelukast and other inhaled drugs taken and their step-downs/step-ups were also considered. The daily dosage of Beclomethasone dipropionate or the equivalent dose of other ICSs (Fluticasone, Budesonide or others) was expressed as low (≤500 mcg), medium (500–1000 mcg) or high (≥1000 mcg), according to GINA classification of ICS dose equivalence [1]. Doses of ICSs and OCs were evaluated at the time of initial and last checks required by the protocol (at least 12 months or more). To diagnose COPD, the following criteria were required: (a) a history of smoking for at least 30 years and no fewer than 15 pack-years, or active smoking for at least 30 years; (b) evidence of emphysema on chest CT scan; (c) post-bronchodilator FEV_1_ < 80% of the predicted value; (d) RV and TLC > 130% of the predicted value; (e) DLCO < 70% of the predicted value (if available); and (f) salbutamol reversibility test < 12%.

The study was approved by Comitato Etico di Area Vasta SUDEST (C.E.A.S.V.E.) of the Azienda USL Toscana Sud-Est at the Azienda Ospedaliera Universitaria Senese (Protocol DUPI, ID:26289; n. 0002892; 19 September 2024).

### Statistical Analysis

The collected data were managed anonymously and aggregated to perform statistical analyses. Continuous variables were expressed as means and standard deviations (SD), while categorical variables were considered as the number of cases and percentages. All continuous variables that had a non-normal distribution are expressed as median values, accompanied by their relative interquartile range (IQR-25° and 75° quartiles). Mann–Whitney and chi square tests were used for comparisons of the various changes obtained after treatment in the two groups. Changes in FEV_1_%, FEF_25–75_%, ACT, exacerbations, ICS doses, and oral corticosteroid dose reduction/withdrawal obtained after Dupilumab were the parameters evaluated. Linear and logistic regression models, adjusted for all confounding factors, were applied to assess whether the anti-IL-4/13 effectiveness was different in SA-COPD compared to SA subjects. Models were adjusted for allergic sensitization to inhalants, rhinitis, sinusitis, nasal polyposis, hypertension, chronic heart disease, gastroesophageal reflux, diabetes, osteoporosis, COPD, obesity, therapy duration (in months), and total serum IgE levels. When necessary, adjustments were also made for pre-treatment FeNO, blood eosinophil counts, lung function, ICS doses, and use of OCs. Linear and logistic regression models, adjusted for all confounding factors, were constructed using stepwise forward selection: models were initiated with one independent variable (SA-COPD compared to SA) and the single most statistically significant variable was iteratively added at each step, continuing until no further additions significantly improved the model’s fit. The finals models contained a maximum of 3 variables; for example, in the one with Δ FEV_1_%, only SA-COPD/SA and BMI remained. Therefore, all of them fit properly.

## 3. Results

Baseline characteristics of the study population are reported in Table 1. Obviously, smoking history and lung function were different. Also, BMI, comorbidities and symptoms were worse in SA-COPD subjects (Table 1).

**Figure 1:**
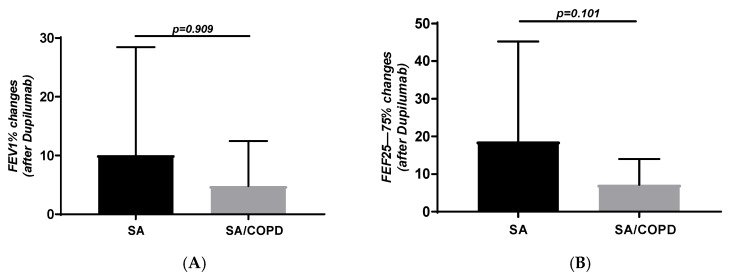
FEV1 (**A**) and FEF25–75 (**B**) changes obtained in subjects with SA and SA-COPD after Dupilumab.

FEV_1_ changes after treatment were 10 ± 18.3% in SA group, whereas they were 4.8 ± 7.6% in individuals with SA-COPD (*p* = 0.909; Figure 1A). Absolute FEV_1_ changes were also similar in the two groups: 0.23 ± 0.59 L in SA and 0.18 ± 0.53 L in SA-COPD (*p* = 0.402). The number of patients with FEV_1_ change ≥ 100/mL after Dupilumab was 20 (60.6%) and 8 (72.7%) in SA and SA-COPD respectively (*p* = 0.737). Moreover, FEF_25–75_% variations after treatment were similar in SA and SA-COPD groups: 18.6 ± 26.5% vs. 7.2 ± 6.8%, respectively (*p* = 0.102; Figure 1B).

**Figure 2:**
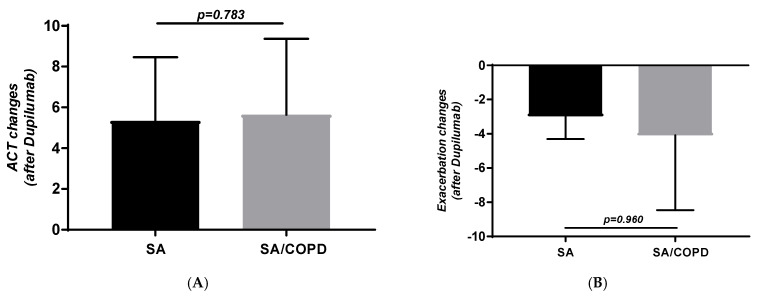
ACT (**A**) and number of exacerbation (**B**) changes obtained in subjects with SA and SA-COPD after Dupilumab.

Similarly, ACT (5.3 ± 3.1 vs. 5.6 ± 3.7; *p* = 0.783; Figure 2A) and exacerbation changes (−2.97 ± 1.3 vs. −4 ± 4.3; *p* = 0.960; Figure 2B) after treatment were similar in the two groups. The number of exacerbations per year after therapy was also similar in the two groups, i.e., 0.25 ± 0.44 in subjects with SA, while it was 0.71 ± 0.5 in those with SA-COPD (*p* = 0.062).

**Figure 3:**
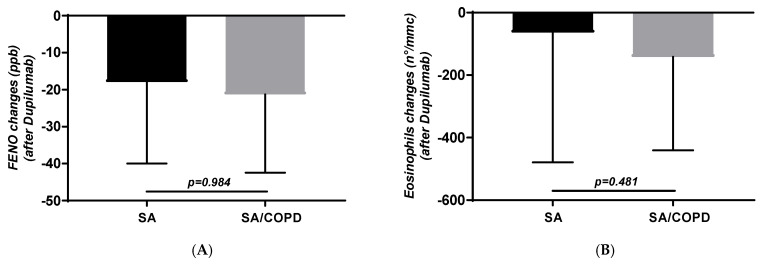
FeNO (**A**) and blood eosinophil (**B**) changes obtained in subjects with SA and SA-COPD after Dupilumab.

No differences were also found in FeNO and BEC changes (−18 ± 22 vs. −21.3 ± 21.1 ppb and −63.6 ± 415 vs. −142 ± 299 cells/µL, respectively, *p* = 0.984 and *p* = 0.481; Figure 3A,B).

**Figure 4:**
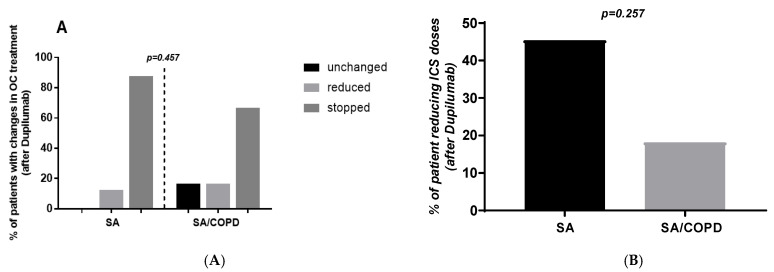
Percentages of patients with changes in OC treatment (**A**) and ICS dose reduction (**B**) obtained in subjects with SA and SA-COPD after Dupilumab.

The percentages of subjects that reduced and stopped OC therapy were 12.5 and 87.5% respectively in the SA group. In contrast, we found that 16.6, 16.6 and 66.7% of individuals did not change, reduced or stopped OC treatment after Dupilumab (*p* = 0.457; Figure 4A) in the group with SA-COPD. The number of subjects that reduced the dose of ICS after treatment was similar in the two populations (45.4 vs. 18.4%; *p* = 0.257; Figure 4B). Post-treatment IgE values were not measured in all patients and were therefore not considered as a possible outcome in assessing the response to Dupilumab.

Linear and logistic regression models, adjusted for all the confounding factors listed above, did not reveal any relationships among the changes in the various outcomes when comparing SA with SA-COPD. β and OR values obtained for various outcomes are reported in Table 2.

## 4. Discussion

Our study, albeit on a limited series of cases, showed the efficacy of Dupilumab in patients with SA-COPD. This treatment led to an improvement that appears to be similar to the one observed in patients with only severe asthma. In fact, the improvement in lung function and symptoms, as well as the reduction in exacerbations, FeNO, and BEC, was similar in patients with SA and those with asthma–COPD overlap (SA-COPD). Moreover, no significant differences were observed between the groups in the reduction in or discontinuation of inhaled or oral corticosteroids (ICSs/OCs). After adjusting for multiple confounding factors, changes in all evaluated outcomes remained comparable between patients with SA-COPD and those with SA treated with dupilumab. These findings suggest that subjects with severe asthma complicated by COPD who exhibit type 2 inflammation may derive similar therapeutic benefits from Dupilumab as patients with severe asthma alone.

There are no studies in the literature relating to Dupilumab and ACO. We found only one study, already cited above, that observed a similar efficacy after 6 months of treatment with various biologics, including Dupilumab, in a group of patients with severe ACO compared to patients with only severe asthma [16]. A recent study confirmed the real-life efficacy of Dupilumab in patients with COPD exhibiting blood eosinophils > 300 cells/uL (a T2 inflammatory pattern), especially in reducing exacerbations and improving symptoms [19], with results comparable to those obtained in our study. As already mentioned, trials in patients with eosinophilic COPD [17,18] have also shown that dupilumab is effective in improving lung function and symptoms and reducing exacerbations. These studies [17,18,19] confirm the results obtained from our analysis, supporting the idea that even patients with severe asthma–COPD overlap with a T2 inflammatory pattern should be treated with biologics, specifically with Dupilumab. Therefore, individuals with severe asthma who have also an associated COPD condition should not be excluded from the opportunity of therapeutic benefits with anti-IL 4/13 treatment. This might concern as many as 27–30% of subjects with severe COPD-associated asthma [2,3] who may benefit from such treatment.

Dupilumab acts in COPD, like in asthma, by blocking the IL-4/IL-13 pathways typical of T2 inflammation, reducing eosinophilia and exacerbations and improving lung function in patients with this specific inflammatory phenotype. Although our patients have typical COPD characteristics, they all express a type 2 inflammatory pattern, where Dupilumab acts most effectively, similarly to the results achieved in asthma alone. Furthermore, by reducing this inflammation, the drug leads to clinical/functional improvement even in patients with only COPD, as demonstrated by recent studies involving subjects with eosinophilic COPD [17,18,19]. Furthermore, about 40% of poorly controlled asthma patients exhibit mucus plugging in high-resolution computed tomography imaging that is associated with worse airflow obstruction, greater type 2 inflammation and more frequent severe exacerbations [20]. A recent randomized controlled trial in patients with uncontrolled asthma showed that dupilumab decreases airway mucus plugging and enhances airway volume and airflow [21]. Since these mucus plugs are linked to a higher risk of COPD exacerbations [22], their reduction/improvement with dupilumab may help explain its effectiveness in COPD.

We do not actually have a standardized definition or diagnostic criteria for ACO. Several definitions have been suggested [23,24,25,26]. As a result, comparing or generalizing findings across studies is difficult because each study uses various definitions and populations. We employed very strict criteria to select patients complicated by COPD, thus making its presence certain, while the presence of asthma is supported by elevated biological markers and allergies (T2 inflammation). This further supports the idea that Dupilumab should also be used in patients with ACO.

The patients with SA-COPD recruited in our study, as required by a strict protocol, exhibit worse characteristics than patients with SA alone (like a higher BMI, a greater number of comorbidities and worse lung function). In fact, symptoms, assessed with ACT, were worse in subjects with SA complicated by COPD. Dupilumab has demonstrated its efficacy even in these patients, which endorses considering biologic treatment, in particular Dupilumab, in ACO. If not treated, these patients might experience more frequent exacerbations and hospitalizations, worse health-related quality of life, and higher healthcare costs than those with the disease alone [2,12,13,14,25].

As already stated, there are no differences among the various parameters measured in the two groups. Looking at our data, we can only hypothesize a possible reduced response with Dupilumab in terms of the improvement in lung function. Indeed, the increase in FEV_1_ and FEF_25–75_ appears to be reduced in SA-COPD, although such a reduction is not statistically significant. When evaluated in a larger number of patients, this difference might be important because COPD subjects, compared to asthmatics, may have less reversible bronchial obstruction as a consequence of smoking. This is confirmed by a recent real-world study that found no benefit on lung function with Dupilumab in COPD patients [19]. This limited functional improvement may be due, as we have already mentioned, to the fixed or poorly reversible obstruction that mostly characterizes the COPD population.

The number of SA-COPD subjects evaluated is small and this could be a limitation of the study. We have to stress that this small sample size could also be the reason for the lack of significance (as compared to SA) in the results, which does not to give strength to our findings, which should be confirmed in larger populations. It must be said that recruiting a large number of participants for this type of research is quite challenging. Indeed, several ACO patients may have not been considered for treatment with a biologic as there was no clear evidence of its efficacy in such subjects. ACO has been excluded from almost all clinical trials of asthma or COPD medications, highlighting the limited evidence for biologic therapy in this subgroup of patients. However, according to our observation, even if on few patients, Dupilumab has also shown to be effective in patients with AS and COPD characteristics. In addition, today, Dupilumab’s usefulness in ACO is supported by trials and a real-life study that have assessed its clinical and functional efficacy in COPD with type T2 inflammation with a high blood eosinophil count [17,18,19]. These studies emphasize the importance of treating type II inflammation even in COPD patients, especially with Dupilumab, as it can provide them with therapeutic benefits. Therefore, considering only COPD and excluding T2 inflammation could be limiting in the treatment of COPD or, in our case, of SA-COPD.

It must also be said that asthma–COPD overlap should be considered a different phenotype from asthma and COPD alone, where Dupilumab, given the potential efficacy demonstrated by our study, could represent a precision therapy for patients with this disease phenotype, especially those with the type 2 inflammatory endotype (allergy/IgE, elevated FeNO and high blood eosinophils). In fact, Dupilumab works precisely by blocking IL-4 and IL-13, two cytokines central to type 2 (T2) inflammation. In COPD, however, not all patients have T2 inflammation: many have neutrophilic or mixed inflammation, which does not respond to this drug.

In conclusion, according to our real-life experience, Dupilumab is effective both in patients with severe asthma alone and in those with severe asthma complicated by COPD. We must always consider T2 inflammation in order to provide the most appropriate treatment for subjects with asthma–COPD overlap. However, further studies on larger case series are needed to confirm these results.

## Figures and Tables

**Table 1:** Characteristics of subjects with severe asthma and those with associated COPD before treatment with Dupilumab.

	Asthma	Asthma/COPD	*p*
N° of subjects [M/F]	33 (75%) (13/20)	11 (25%) (7/4)	**0.0001**
Age	57.8 ± 9.8	63.9 ± 13.2	0.118
BMI	25.9 ± 4.2	29.7 ± 4.2	**0.015**
Smokers/ex-smokers (n. of patients)	11 (33.3%)	11 (100%)	**0.055**
Packs/year	0 (0–10)	30 (20–40)	**0.0001**
Age of asthma onset	29 (12–47)	44 (12–50)	0.310
Pre-biologic FeNO (ppb)	39 (17–50.5)	39.5 (9.5–60.5)	0.848
Pre-treatment FEV_1_% predicted	69 (52–101.5)	60 (45–73)	**0.059**
Pre-treatment FEF_25–75_% predicted	38 (21–93)	40 (18–48)	0.551
Pre-treatment RV% predicted	98 (82–124)	147 (134–175)	**0.0001**
Pre-treatment TLC% predicted	98 (88–105)	131 (100–148)	**0.0025**
Pre-treatment DLCO% *(evaluated in 13 patients in SA and 9 in SA/COPD)*	84 (80–88.5)	65 (49–73)	**0.001**
Pre-treatment bronchodilation reversibility test with salbutamol	9 (5–13)	4 (2–11)	**0.088**
Monosensitized (to 1 allergen)	10 (30.3%)	3 (27.3%)	0.912
Polisenitized (to ≥2 allergens)	15 (45.4%)	5 (45.4%)	
Baseline blood eosinophil counts (cells/µL)	380 (280–575)	400 (200–700)	0.984
Baseline total serum IgE (UI/mL)	153 (80–465.8)	324 (210–575)	**0.049**
Comorbidities n. 0–1 (n. of patients)	25 (57.1%)	8 (59.4%)	**0.055**
Comorbidities n. ≥ 2 (n. of patients)	8 (42.8%)	3 (40.6%)	
Sensitizations to at least one aeroallergen (n. of patients)	23 (69.7%)	7 (63.6%)	0.656
Pre-treatment ACT	16 (13.5–18)	12 (10–15)	**0.013**
Pre-treatment n. of exacerbations/year	3 (2–4)	3 (3–10)	0.219
Rhinitis (n. of patients)	24 (72.7%)	6 (54.5%)	0.779
Chronic rhinosinusitis/nasal polyposis (n. of patients)	23 (69.7%)	6 (54.5%)	0.781
N° of patients on OC treatment before biologic	8 (24.2%)	6 (54.5%)	0.311
Months of biologic treatment (ΔT0–T1)	12 (12)	12 (12–24)	**0.087**

Table data are presented as mean and standard deviation (SD) or as median and interquartile range [IQR].

**Table 2:** Linear (β) and logistic (OR) regression models comparing subjects with SA and SA-COPD on clinical/functional outcomes.

*Linear Regression Models*	β [95% CI]	*p*
∆ FEV_1_%	−1.018 [−13.120–11.084]	0.866
∆ FEF_25–75_%	−7.704 [−22,382–6975]	0.293
∆ ACT	0.198 [−2051–2446]	0.860
∆ Exacerbations	0.101 [−1688–1891]	0.909
∆ FENO	−9.100 [−23,365–5164]	0.205
∆ Eosinophils	−78.364 [−353,225–196,498]	0.568
***Logistic Regression Models***	**OR [95% CI]**	
ICS dose reductions (*vs. unchanged ICS doses*)	0.674 [0.081–5.602]	0.715
OC dose reductions or discontinuation (*vs. unchanged OC treatment*)	0.03 [0.000–3.065]	0.102

FEV_1_%, FEF_25–75_%, ACT, exacerbation changes, ICS dose reductions (vs. unchanged doses), and oral corticosteroid (OC) dose reductions or discontinuations (vs. unchanged OC treatment) were analyzed. All models were adjusted for allergic sensitization to inhalants, rhinitis, sinusitis, nasal polyposis, hypertension, chronic heart disease, gastroesophageal reflux, diabetes, osteoporosis, COPD, obesity, therapy time (months), and total serum IgE and blood eosinophils, as well as, when necessary, for lung function, doses of ICSs and use of OCs.

## Data Availability

All data generated or analyzed during this study are included in this article. Further inquiries can be directed to the corresponding author.
